# The Anisotropic Thermal Expansion of Non-linear Optical Crystal BaAlBO_3_F_2_ Below Room Temperature

**DOI:** 10.3389/fchem.2018.00252

**Published:** 2018-06-28

**Authors:** Xingxing Jiang, Naizheng Wang, Maxim S. Molokeev, Wei Wang, Shibin Guo, Rongjin Huang, Laifeng Li, Zhanggui Hu, Zheshuai Lin

**Affiliations:** ^1^Technical Institute of Physics and Chemistry, Chinese Academy of Sciences, Beijing, China; ^2^University of the Chinese Academy of Sciences, Beijing, China; ^3^Laboratory of Crystal Physics, Kirensky Institute of Physics, Federal Research Center KSC SB RAS, Krasnoyarsk, Russia; ^4^Department of Physics, Far Eastern State Transport University, Khabarovsk, Russia; ^5^Department of Engineering Physics and Radioelectronic, Siberian Federal University, Krasnoyarsk, Russia; ^6^Key Laboratory of Cryogenics, Technical Institute of Physics and Chemistry, Chinese Academy of Sciences, Beijing, China; ^7^Institute of Functional Crystals, Tianjin University of Technology, Tianjin, China

**Keywords:** BABF, anisotropic thermal expansion, phonon stimulation, NLO optical property, low temperature

## Abstract

Thermal expansion is a crucial factor for the performance of laser devices, since the induced thermal stress by laser irradiation would strongly affect the optical beam quality. For BaAlBO_3_F_2_ (BABF), a good non-linear optical (NLO) crystal, due to the highly anisotropic thermal expansion its practical applications are strongly affected by the “tearing” stress with the presence of local overheating area around the laser spot. Recently, the strategy to place the optical crystals in low-temperature environment to alleviate the influence of the thermal effect has been proposed. In order to understand the prospect of BABF for this application, in this work, we investigated its thermal expansion behavior below room temperature. The variable-temperature XRD showed that the ratio of thermal expansion coefficient between along *c*- and along *a(b)*- axis is high as 4.5:1 in BABF. The Raman spectrum combined with first-principles phonon analysis revealed that this high thermal expansion anisotropy mainly ascribe to progressive stimulation of the respective vibration phonon modes related with the thermal expansion along *a(b)*- and *c*-axis. The good NLO performance in BABF can be kept below room temperature. The work presented in this paper provides an in-depth sight into the thermal expansion behavior in BABF, which, we believe, would has significant implication to the manipulation in atomic scale on the thermal expansion of the materials adopted in strong-field optical facility.

## Introduction

Thermal expansion behavior is a crucial performance factor for materials used in lasers, due to its affinity with the ability of the optical outputting (Wynne et al., 1999; Wang et al., 2007; Mangin et al., 2011; Ito et al., 2017; Fang et al., 2018). Non-linear optical (NLO) crystal, a type of crystals for laser frequency conversion, has played a key role in the broadening of laser spectrum and become one of the most prevailing branch of optical materials (Chen et al., 1999, 2012; Cyranoski, 2009; Meng et al., 2009; Lin et al., 2014). Among the commercial NLO crystals, BaAlBO_3_F_2_(BABF) crystal, is a very excellent member, and exhibit many superiority over its congeners in the output of laser in 532 and 355 nm (Zhou et al., 2009; Hu et al., 2011; Yue et al., 2011; Yang et al., 2016). As for the practical application of BABF, the optical spot with high-power is usually focused on one point of the crystal, and thus “tearing” stress resulted from anisotropic thermal expansion would severely affect the beam quality and outputting power. The previous study about the thermal property of BABF revealed that above room temperature, its thermal expansion coefficient along *c*-axis is about eight times of that along *a(b)-axis*, and the strongly anisotropic thermal expansion has been the major disadvantage to restrict its practical application (Yue et al., 2011). However, the microscopic mechanism of the thermal expansion anisotropy in BABF has not been investigated. Recently, it is proposed that the thermal effect on the performance of some optical crystal used in laser can be eliminated to some extent below room temperature (Marrazzo et al., 2016; Veselov et al., 2016; Roitero et al., 2018), which provide a feasible method to overcome the problem about the thermal effect. However, the thermal expansion behavior of BABF under low temperature has not been investigated yet, and its performance index below room temperature remain unclear. Therefore, it is desirable to perform a study about the thermal expansion to give a comprehensive evaluation on the application prospect below room temperature and elaborate the structure-property relationship of the anisotropic thermal expansion of BABF.

In this work, using variable-temperature X-ray diffraction, the thermal expansion behavior of BABF below room temperature is studied, and the mechanism of the thermal expansion behavior is elaborated by Raman spectrum and first-principles phonon analysis. The optical property below room temperature is also investigated by first-principles calculation. It is elucidated that BABF still exhibit a relatively strong anisotropic thermal expansion below room temperature, but its anisotropy is less prominent than that above room temperature. The optical performance is also slightly improved under low temperature. These result indicates that the performance below room temperature for the laser generation is superior than that above room temperature, and BABF is a potential NLO crystals in cryogenic system.

## Experimental and computational method

### Sample preparation

Polycrystalline BABF was synthesized through solid-state reaction. Analytically pure BaF_2_, Al_2_O_3_, and B_2_O_3_ in stoichiometric ratio as the starting materials were mixed homogeneously by agate mortar. The well-grinded reactant then was placed into muffle furnace and was heated gradually up to 800°C at the rate of 0.5°C/min with several intermediate careful grinding at 300, 600, and 800K. After cooling to room temperature, the white powder of target compound was obtained.

### Variable temperature X-ray diffraction

Variable temperature X-ray diffraction patterns were recorded from 13 to 300K with the internal of 20K. Each pattern was recorded with Bruker D8 advanced X-ray diffractometer Cu Kα radiation (Kα1 = 1.5406Å and Kα2 = 1.5443Å) on the finely grounded powder samples. The angular scanning range were set to 10–90° with a step of 0.01° and scanning rate 0.5 s/step. The crystal structures under different temperature were refined by Rietveld method (Rietveld, 1969) using TOPAS 4.2 program (BAXS, 2008). Based on the refined cell parameters. The thermal expansion coefficient was fitted by the PASCal software (Cliffe and Goodwin, 2012).

### Raman spectrum

The Raman pattern was recorded from 100 to 1,500 cm^−1^ at room temperature, using in Via-Reflex, equipped with a solid state laser with a wavelength of 532 nm. In order to improve the signal to noise ratio of the spectra, 10 integrations were carried out with an integration time of 10 s at a nominal resolution of 1 cm^−1^ and a precision of 1 cm^−1^.

### First-principles calculation

The first-principles calculation were performed by CASTEP (Clark et al., 2005), a plane-wave pseudopotential total energy package based on density functional theory (DFT) (Kohn and Sham, 1965; Payne et al., 1992). The functionals developed by Perdew, Burke, and Ernzerhof (PBE) (Perdew et al., 1996) in generalized gradient approximation (GGA) (Perdew and Wang, 1992) form were adopted to describe the exchange-correlation energy. The optimized norm-conserving pseudopential (Hamann et al., 1979) in (Kleinman and Bylander, 1982) form were used to model the effective interaction between the valcence electrons and atom cores, which allow us to use a small plane basis set without compromising the accuracy required by the calculation. High kinetic energy cutoff of 900 eV and Monkhorst-pack (Monkhorst and Pack, 1976) *k*-point mesh spanning less than 0.04 Å^−1^ in the Brillouin zone were chosen. The vibrational property was calculated by linear response formalism (Baroni et al., 2001), in which the phonon frequencies were obtained by the second derivative of the total energy with respect to a given perturbation. The band gaps at variable temperature were predicted by hybridized functionals PBE0 based on the refined structure at respective temperature, and the refractive index and SHG coefficients were calculated by Kramers–Kronig transform based on the electronic transition matrix (PED, 1985) and the software developed by our group based on length-gauge formalism (Lin et al., 1999, 2014).

## Result and discussion

**Figure 1:**
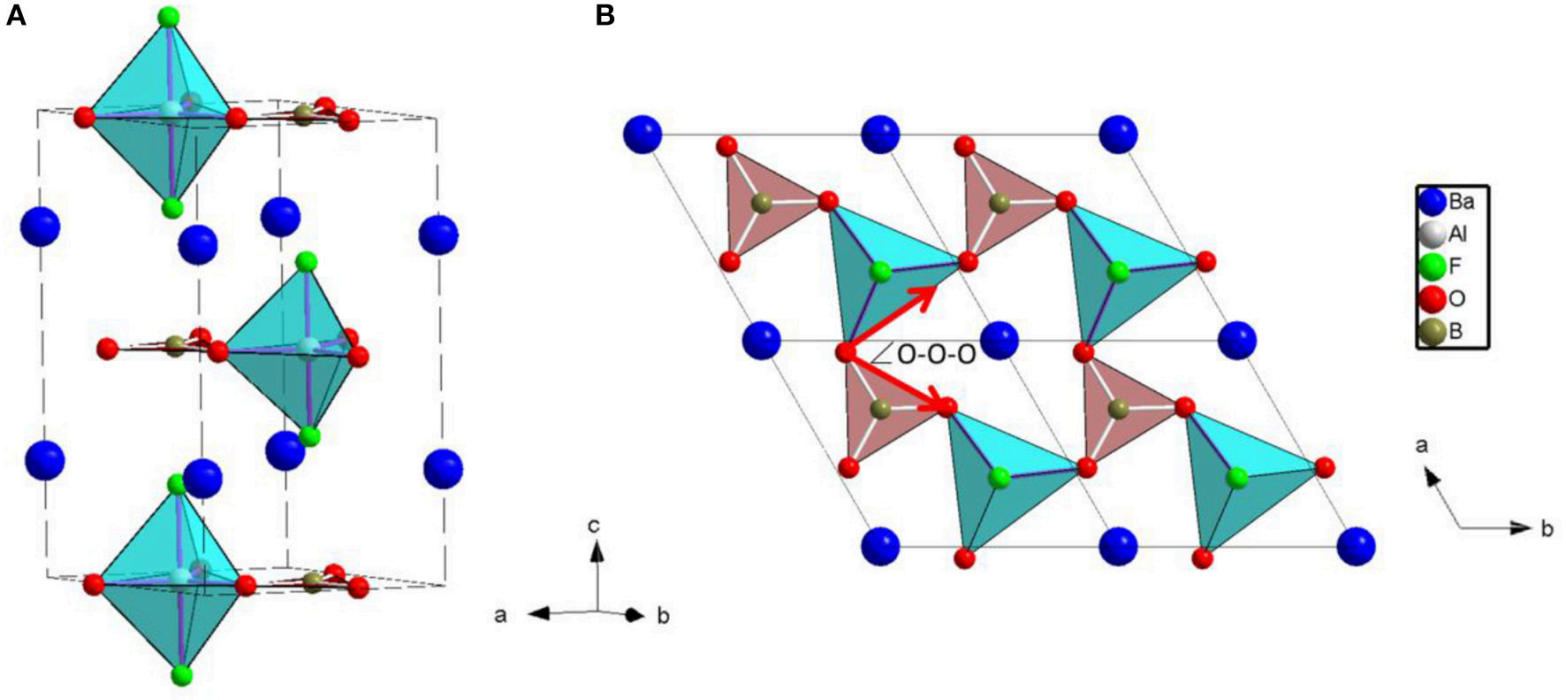
Crystal structure of BaAlBO_3_F_2_ viewed along **(A)** *(a,b)* plane and **(B)** *c*-axis. The BO_3_ and AlO_3_F_2_ groups are represented by pink triangles and light blue trigonal bipyramids, respectively.

The crystal structure of BABF was determined by Hu et al. (2011). BABF possesses a hexagonal lattice with layered-tendency structure, as displayed in Figure 1. One boron atom is bonded with three oxygen atoms to form a planar BO_3_ triangles, and one aluminum atom coordinates with three oxygen and two fluorine atoms to generate the AlO_3_F_2_ trigonal bipyramid. The BO_3_ triangles and AlO_3_F_2_ trigonal bipyramids are aligned alternatively in the ratio 1:1 by sharing the vertical oxygen atoms, giving rise to the infinite two-dimensional [AlBO_3_F_2_]_∞_ layer, in which the BO_3_ triangle and the AlO_3_ base of AlO_3_F_2_ trigonal bipyramids are aligned absolutely parallel to *(a, b)* plane. [AlBO_3_F_2_]_∞_ layer are further connected with each other via the Coulomb interaction between the dangling fluorine atoms and interstitial barium atoms to generate the layered-tendency structure.

**Figure 2:**
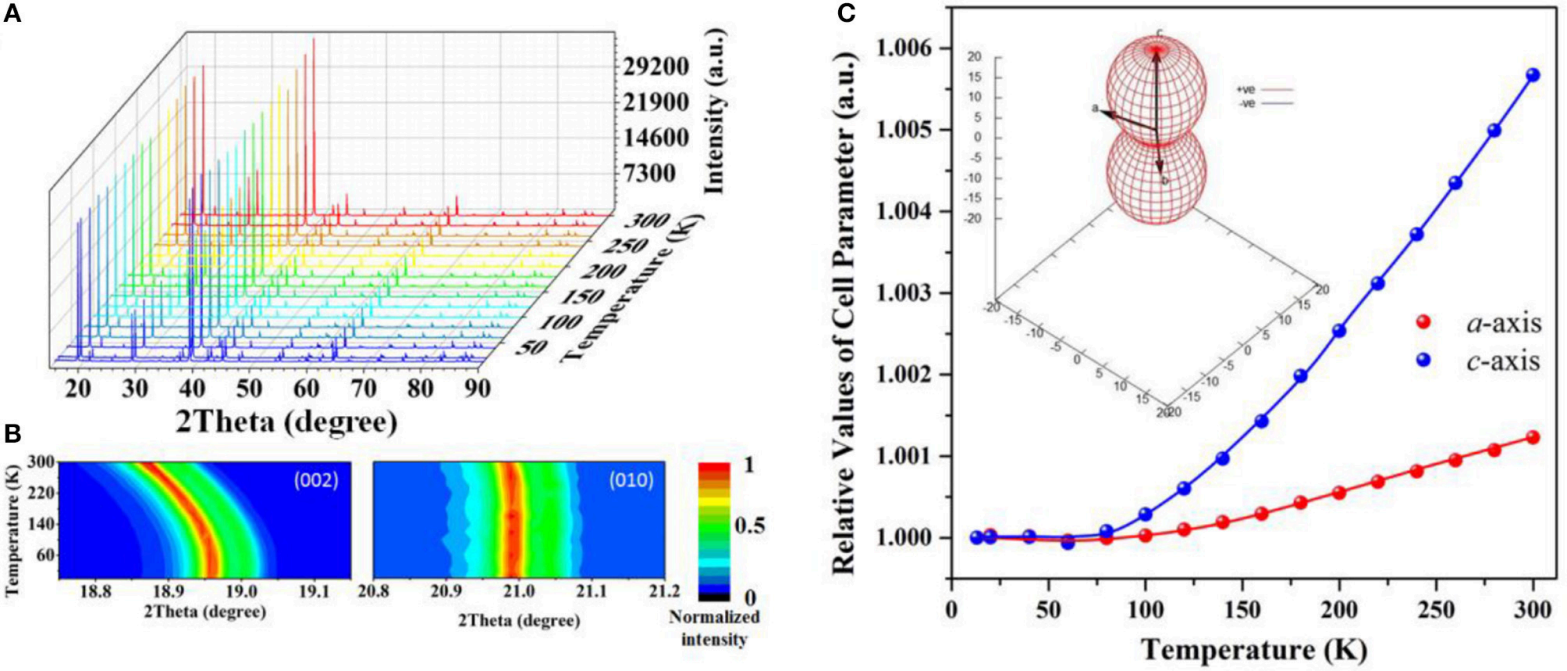
Result of VT-XRD on BABF: **(A)** the VT-XRD patterns, **(B)** 2θ-temperature contour map of the diffraction peak of (002) and (010) plane and **(C)** evolution of cell parameters with respect to temperature. The insert in **(C)** displays the special distribution of thermal expansion coefficient plotted by PASCal software (Cliffe and Goodwin, 2012).

The variable-temperature X-ray diffraction (VT-XRD) patterns are plotted in Figure 2A. It is observed that no new peaks appear as temperature elevate from 13 to 300K, indicating the absence of structural phase-transition and manifesting its high thermodynamical stability. The temperature-deduced diffraction peak-shifting of the lattice plane corresponding to *c*-axis is much more prominent than that of *a(b)-axis* (Figure 2B), suggesting the high anisotropic thermal expansion in BABF. According to the cell parameters extracted from the VT-XRD by Rietveld refinement (Figure S1, Figure 2C and Table S1), the average thermal expansion coefficient are 4.42(39)/MK and 20.08(71)/MK along *a(b)-axis* and *c*-axis respectively, which confirmed that the thermal expansion along *c*-axis is much more prominent than that along *a(b)*-axis. More importantly, the ratio (4.5:1) between the thermal expansion coefficients along *c*- and *a(b)-axis* below room temperature is much lower than that above room temperature (7.6:1), and the low anisotropy of the thermal expansion below room temperature is more favorable for elimination of the stress induced by temperature gradient when irradiated by laser. Additionally, It should be emphasized that at low temperature (13–100K) the *a(b)*-axis exhibit very low thermal expansion with the fitted linear thermal expansion coefficient 0.11(25)/MK, which is lower than that of *c*-axis [2.44(98)/MK] by one order and can be categorized to typical zero thermal expansion.

**Figure 3:**
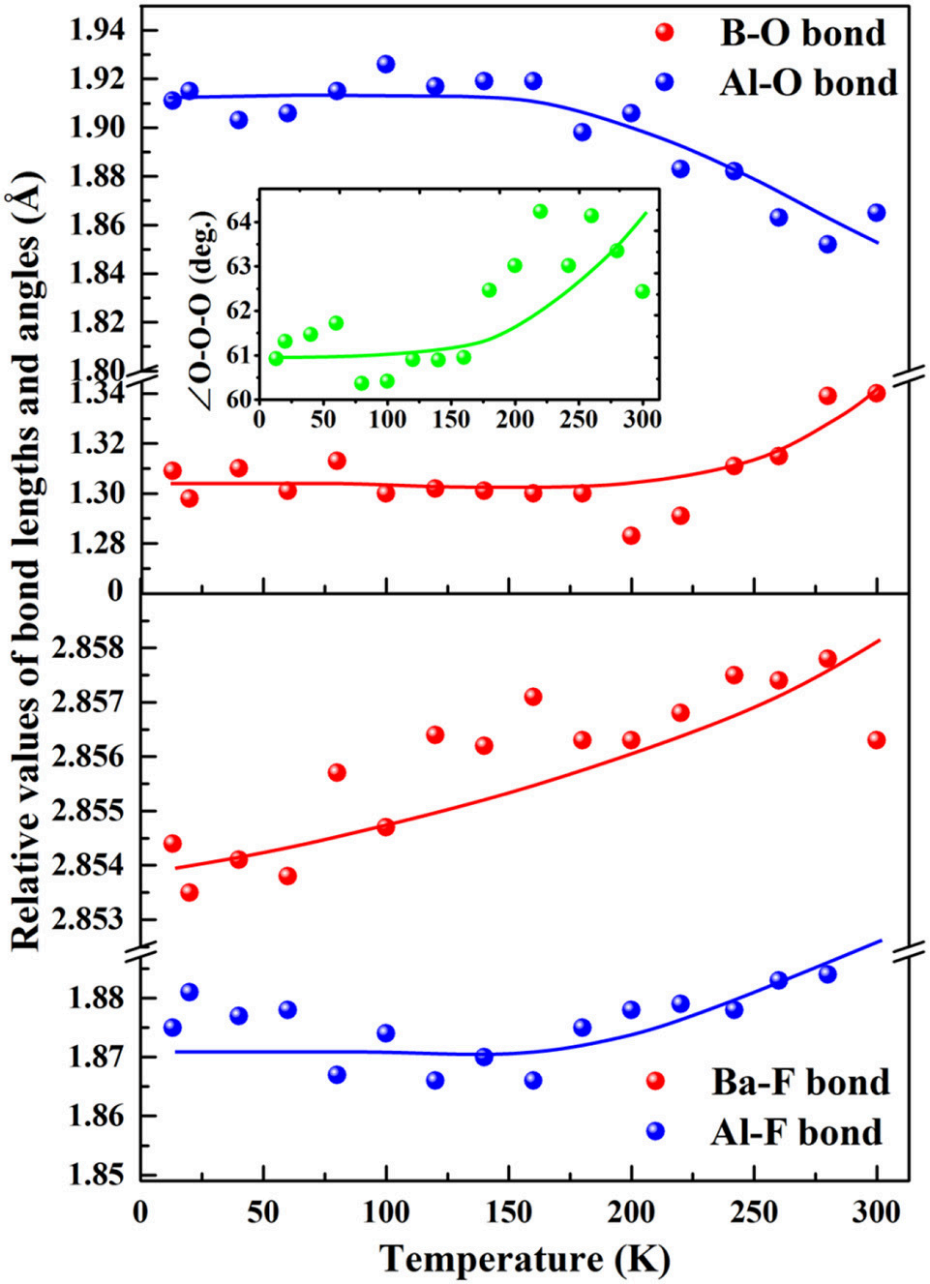
Function of refined bond length and angles with respect to temperature in BABF.

The most intuitive way to investigate the mechanism of the thermal expansion is to trace the modification of bond length and angles with respect to temperature. The *a(b)*-axis is exclusively determined by B-O, Al-O bonds, and ∠ O-O-O angle, while *c*-axis is dominantly determined by the Ba-F and Al-F bond. According to the temperature-dependent bond length and angles (Figure 3 and Table S2), despite the clutter of the point due to the difficulty to exactly determine the position of the constituted light atoms in BABF, a roughly changing tendency can be observed. Below 140K the bond length and angles accounting for the change of *a(b)*-axis almost keep constant, resulting in the rigidity of *a(b)*-axis under low temperature. And dramatic modification only occur above 140K: B-O bond, and ∠ O-O-O angle increase from 1.301 to 1.340Å (by 3%) and 60.892–62.437° (by 2.5%) respectively as temperature increase from 140K to 300K, which both positively contribute to the thermotropic expansion. Extruded by the increase of ∠ O-O-O angle, Al-O bond decrease from 1.919 to 1.865Å (by 2.8%), which slightly cancel out the expansion effect originated from the increase of B-O bond and ∠ O-O-O angle, leading to the relative low thermal expansion along *a(b)*-axis. On the contrary, both Al-F and Ba-F bond increase as temperature increase, and thus *c*-axis exhibit a normal thermal expansion ~10/MK of inorganic crystals.

**Figure 4:**
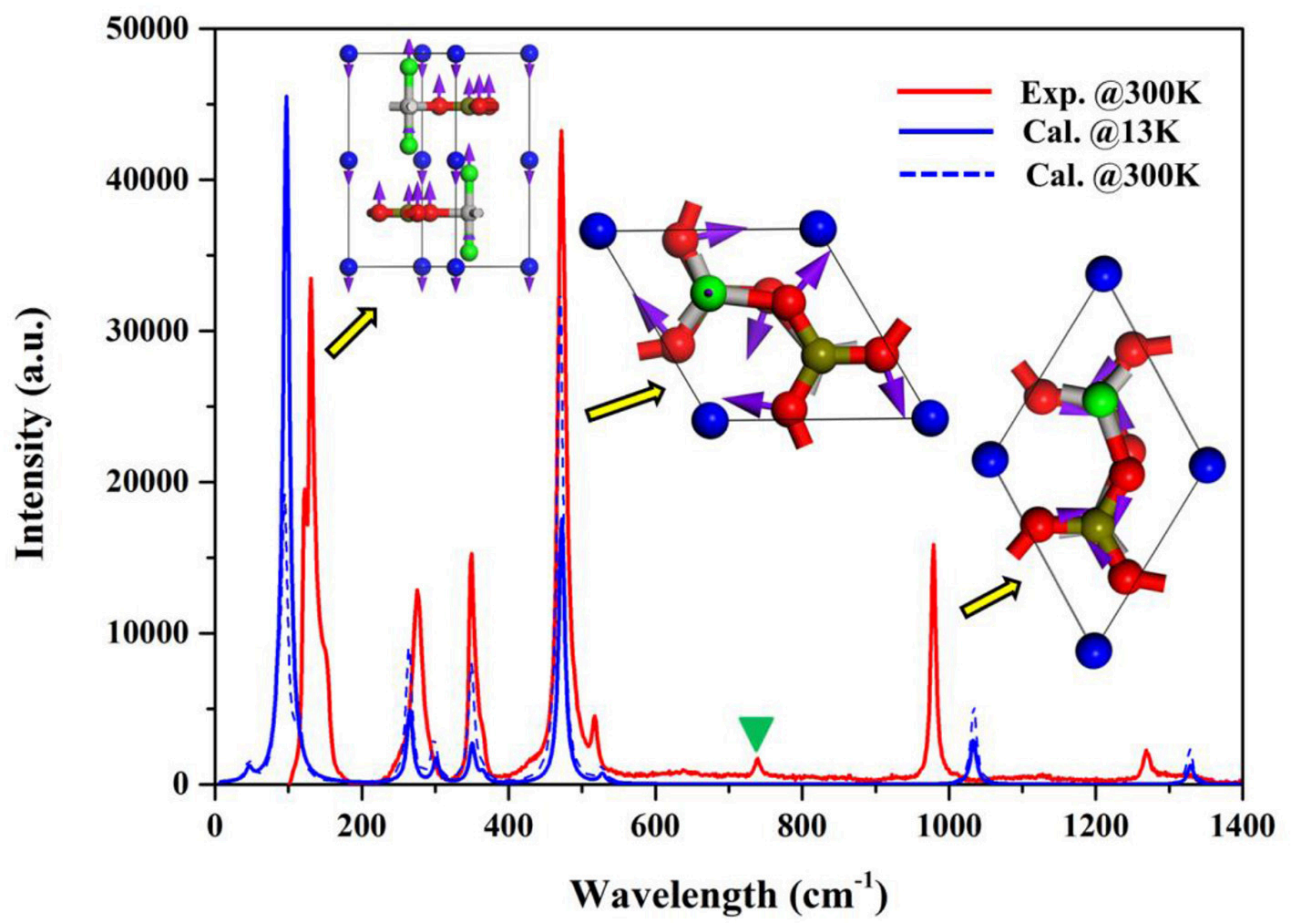
Measured and calculated Raman spectrum of BABF. The modes with the highest peaks at 87, 473, and 1,030 cm^−1^ are used to schematically describe the atomic vibration of type-I, II, and III phonon modes respectively. The peaks at around 720cm^−1^ labeled by green triangle in the experimental spectrum is attributed to the impurity.

The thermal expansion of materials is closely related with the temperature-induced stimulation of lattice vibration, and it is anticipated that first-principles phonon mode assignment would compensate the disadvantage of the littery temperature-dependent bond length and angles and make the mechanism of the thermal expansion in BABF more clear. The irreducible representation of P-62c space group at Γ-point yields a sum of 30E+7A1+8A2 phonon modes (see Table S3). According to the calculated Raman spectrum (Figure 4), six principal peaks are observed at around 87, 266, 338, 473, 1,030, and 1,331cm^−1^ respectively. The calculated Raman spectrum is in rather agreement with the measured ones, verifying the accuracy of the computational method. According to the vibrational vector projected onto the real space of respective phonon modes, it is revealed that as frequency increase the phonon roughly experience a evolution from the type-I (mode 1–7), -II (mode 8–29) to -III (mode 30–45). In type-I, the configuration of 2D [AlBO_3_F_2_]_∞_ layer keep constant and it vibrate as a rigid unit along *c*-axis with the reverse phase with that of interstitial barium atoms. This indicates that at low temperature the vibration modes related with the stretch between [AlBO_3_F_2_]_∞_ and barium would be firstly stimulated, in which the size of [AlBO_3_F_2_]_∞_ layer along *a(b)*-axis remain constant. Therefore, below 100K, BABF exhibit slight thermal expansion along *c*-axis and rigidity along *a(b)*-axis. In type-II, the oxygen atoms within [AlBO_3_F_2_]_∞_ layer vibrate along the direction almost vertical to B-O bonds. This implies that in these modes, the BO_3_ triangles rotate within *(a,b)* plane as the rigid units. This effect corresponds to the slight contraction of Al-O bond in AlO_3_F_2_ trigonal bipyramids and also would lead to the expansion of the interspace within [AlBO_3_F_2_]_∞_ layer, which eventually lead to the weak thermal expansion along *a(b)-*axis. Meanwhile, the stretch vibration of Al-F bonds also partially account for the modes of type-II, which enhanced the thermal expansion along *c*-axis. In type-III modes, the phonon vibration mainly originate from the stretch of B-O bonds, which is related with the elongation of B-O bonds. It should be emphasized that high temperature is required to stimulate these vibration modes (such as ~ 1300K for the mode at 1030 cm^−1^), and these modes can only afford the thermal expansion at high temperature and almost contribute nothing to that below 300K. Moreover, as temperature increase, all the Raman peaks are red-shifted, affording the normal general thermal expansion. Besides, it should be emphasized that the weight of modes related with the thermal expansion along *a(b)*-axis at 300K is much more prominent than that at 13K, which also result in the enhanced thermal expansion along *a-(b)* axis as temperature increase. Therefore, the anisotropic thermal expansion behavior in BABF is mainly attributed to the progressive stimulation of the respective vibration modes related with the expansion of *c*-axis to that of *a(b)*-axis. Moreover, it should be emphasized that as temperature increase, the amplitude of atomic vibration would increase, and the phonon anharmonicity would be enhanced. Since the thermal expansion is originated from phonon anharmonicity, the thermal expansion of both *a*-axis [4.42(39)/MK] and *c*-axis [20.08(71)/MK] between 13 and 300 are less prominent than that those between 303 and 1,073K [6.3 and 48.1/MK, respectively (Yue et al., 2011)].

**Figure 5:**
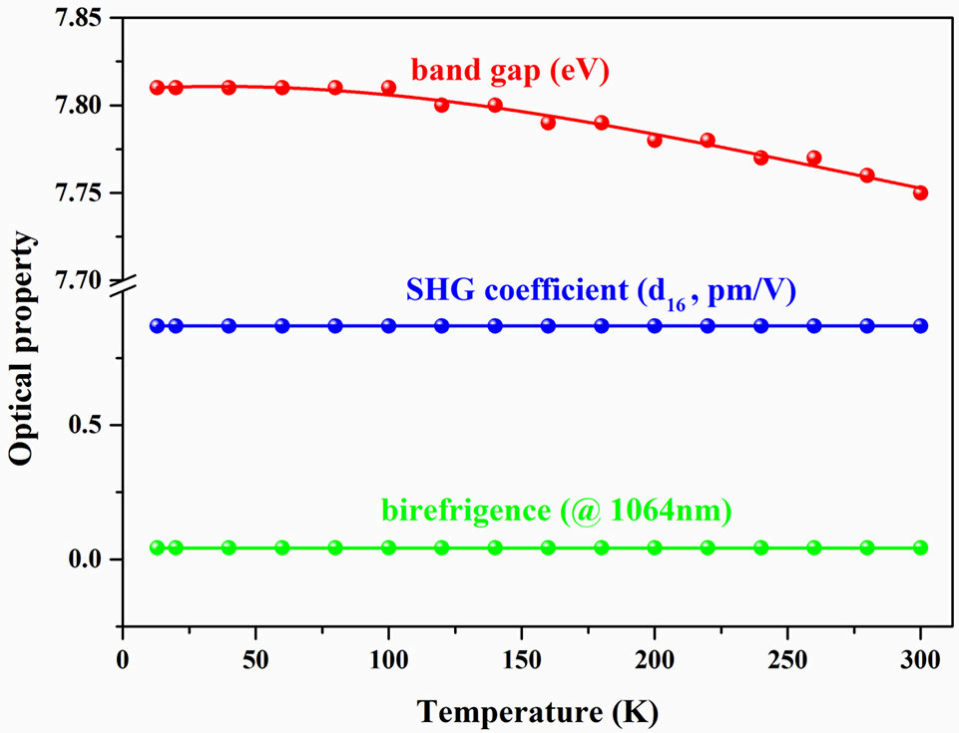
Function of the calculated band gap, SHG coefficient and birefringence (@1,064 nm) of BABF with respect to temperature.

Additionally, considering that the optical performance is also crucial to the usage in apparatus operated at low temperature, the temperature-dependent optical property was also studied based on the refined structure by first-principles calculation (Figure 5 and Table S4). Accordingly, both the SHG coefficient and birefringence present ultra-stability under temperature fluctuation, and the band gaps are broadened as temperature decrease, which guarantee the optical transmittance of BABF below room temperature. All these observation elucidated that the good optical performance of BABF can be well-kept, which is favorable to its practical application in the apparatus operated below room temperature.

## Conclusion

The thermal expansion behavior below room temperature of BABF, an important ultraviolet NLO crystal, is investigated. It is revealed that the BABF exhibit relatively high anisotropic thermal expansion, and the near-zero thermal expansion behavior along *a(b)*-axis below 100K was observed. Based on the refined temperature-dependent crystal structure, Raman spectrum and first-principles vibration phonon analysis, it is elucidated that the anisotropic thermal expansion behavior in BABF mainly stem from the progressive stimulation of the phonon modes related with the thermal expansion along *a(b)*- and *c*-axis. Moreover, it is revealed that the optical performance of BABF can also be well-kept under low temperature, which is very favorable for its practical application. Our study exhibit a comprehensive investigation about the thermal and optical property below room temperature and shed light on the structural origin of the thermal expansion of BABF. We believed that the conclusion deduced from our study further deepens the understanding about the performance of BABF and would be beneficial for its practical application under complex environment.

## Author contributions

XJ, NW, and MM performed the experiment. XJ performed the first-principles simulation. WW, SG, RH, and LL help in the variable X-ray diffraction. ZL, ZH, and XJ written the manuscript.

### Conflict of interest statement

The authors declare that the research was conducted in the absence of any commercial or financial relationships that could be construed as a potential conflict of interest.
