# Supplementary material for: The Anisotropic Thermal Expansion of Non-linear Optical Crystal BaAlBO3F2 Below Room Temperature
Source: Front Chem. 2018 Jun 28;6:252. doi: 10.3389/fchem.2018.00252 (PMC6033077; doi:10.3389/fchem.2018.00252)
Supplement: Supplementary file 1 [file Data_Sheet_1.DOCX]

**Supporting information for**

**The anisotropic thermal expansion of nonlinear optical crystal BaAlBO_3_F_2_ below room temperature**

Xingxing Jiang, ^1,5^ Naizheng Wang, ^1,5^ Maxim S. Molokeev,^2,6,7^ Wei Wang, ^3^ Shibin Guo, ^3^ Rongjin Huang^3^, Laifeng Li, ^3^ Zhanggui Hu*,^4^ and Zheshuai Lin*^,1,5^

^1^Technical Institute of Physics and Chemistry, Chinese Academy of Sciences, Beijing 100190, China. *e-mail: zslin@mail.ipc.ac.cn.

^2^Laboratory of Crystal Physics, Kirensky Institute of Physics, Federal Research Center KSC SB RAS, Krasnoyarsk 660036, Russia.

^3^Key Laboratory of Cryogenics, Technical Institute of Physics and Chemistry, Chinese Academy of Sciences, Beijing 100190, China.

^4^Institute of Functional Crystals, Tianjin University of Technology, Tianjin 300384, PR. China. *email: hu@mail.ipc.ac.cn

^5^University of the Chinese Academy of Sciences, Beijing 100049, China.

^6^Department of Physics, Far Eastern State Transport University, Khabarovsk 680021, Russia.

^7^ Siberian Federal University, Krasnoyarsk 660041, Russia.

Table S1 the refined cell parameter at various temperature of BABF.

|  | a | c |
| --- | --- | --- |
| 13 | 4.878871(41) | 9.346052(62) |
| 20 | 4.879024(40) | 9.346053(64) |
| 40 | 4.878972(46) | 9.346145(64) |
| 60 | 4.878695(43) | 9.345456(66) |
| 80 | 4.878867(44) | 9.346757(68) |
| 100 | 4.879006(41) | 9.348686(67) |
| 120 | 4.879348(42) | 9.351669(68) |
| 140 | 4.879794(47) | 9.355059(83) |
| 160 | 4.880303(45) | 9.359354(96) |
| 180 | 4.880971(43) | 9.364584(77) |
| 200 | 4.881543(44) | 9.369720(69) |
| 220 | 4.882204(48) | 9.375164(70) |
| 240 | 4.882819(41) | 9.380769(72) |
| 260 | 4.883485(42) | 9.386628(67) |
| 280 | 4.884099(42) | 9.392663(85) |
| 300 | 4.884868(45) | 9.399089(74) |

Figure S1 the refinement plot for the XRD patterns at 13K and 300K of BABF


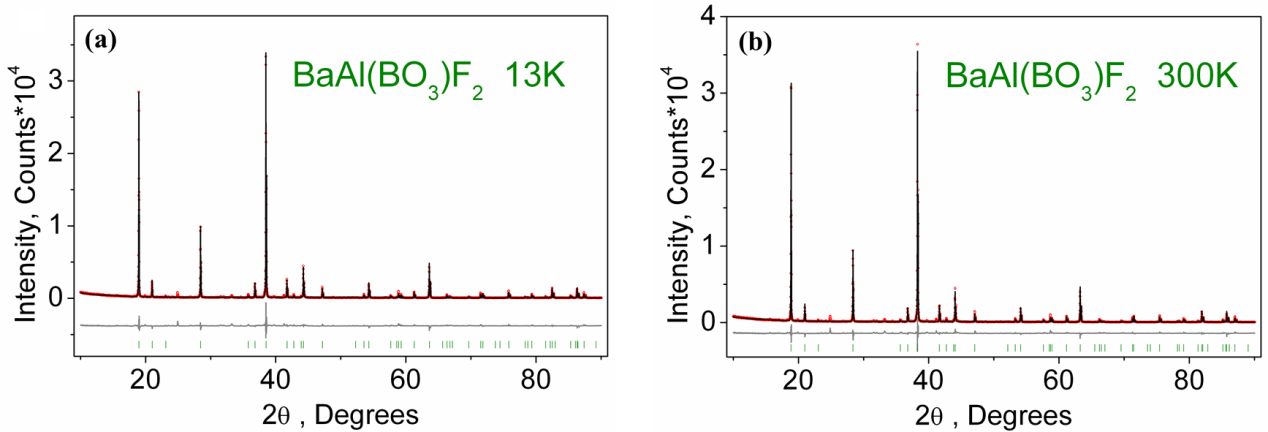


Table S2 the refined cell parameter at various temperature of BABF.

| T,K | d(B-O) | d(Al-O) | ∠O-O-O | Ba-F | d(Al-F) |
| --- | --- | --- | --- | --- | --- |
| 13 | 1.309 | 1.911 | 60.927 | 2.8544 | 1.875 |
| 20 | 1.298 | 1.915 | 61.317 | 2.8535 | 1.881 |
| 40 | 1.310 | 1.903 | 61.471 | 2.8541 | 1.877 |
| 60 | 1.301 | 1.906 | 61.720 | 2.8538 | 1.878 |
| 80 | 1.313 | 1.915 | 60.363 | 2.8557 | 1.867 |
| 100 | 1.300 | 1.926 | 60.413 | 2.8547 | 1.874 |
| 120 | 1.302 | 1.917 | 60.906 | 2.8564 | 1.866 |
| 140 | 1.301 | 1.919 | 60.892 | 2.8562 | 1.870 |
| 160 | 1.300 | 1.919 | 60.954 | 2.8571 | 1.866 |
| 180 | 1.300 | 1.898 | 62.464 | 2.8563 | 1.875 |
| 200 | 1.283 | 1.906 | 63.014 | 2.8563 | 1.878 |
| 220 | 1.291 | 1.883 | 64.235 | 2.8568 | 1.879 |
| 240 | 1.311 | 1.882 | 63.016 | 2.8575 | 1.878 |
| 260 | 1.315 | 1.863 | 64.139 | 2.8574 | 1.883 |
| 280 | 1.339 | 1.852 | 63.346 | 2.8578 | 1.884 |
| 300 | 1.340 | 1.865 | 62.437 | 2.8563 | 1.898 |

Table S3 the calculated wavelength, irreducible representation, IR/Raman activity of the phonon modes at Γ-point of Brillouin zone in BABF

|  | Wavelength  (cm^-1^) | Irreducible representation | IR active | Raman active |
| --- | --- | --- | --- | --- |
| 1 | 47 | E | N | Y |
| 2 | 47 | E | N | Y |
| 3 | 66 | A2 | Y | N |
| 4 | 87 | E | Y | Y |
| 5 | 87 | E | Y | Y |
| 6 | 88 | E | Y | Y |
| 7 | 88 | E | Y | Y |
| 8 | 94 | A2 | N | N |
| 9 | 98 | E | N | Y |
| 10 | 98 | E | N | Y |
| 11 | 112 | A1 | N | N |
| 12 | 117 | E | N | Y |
| 13 | 117 | E | N | Y |
| 14 | 246 | E | Y | Y |
| 15 | 246 | E | Y | Y |
| 16 | 266 | E | Y | Y |
| 17 | 266 | E | Y | Y |
| 18 | 302 | A1 | N | Y |
| 19 | 321 | A2 | N | N |
| 20 | 338 | A2 | Y | Y |
| 21 | 351 | E | N | Y |
| 22 | 351 | E | N | Y |
| 23 | 366 | E | N | Y |
| 24 | 366 | E | N | Y |
| 25 | 372 | A1 | N | N |
| 26 | 382 | A2 | Y | N |
| 27 | 427 | A1 | N | N |
| 28 | 473 | A1 | N | Y |
| 29 | 474 | A2 | N | N |
| 30 | 528 | E | Y | Y |
| 31 | 528 | E | Y | Y |
| 32 | 529 | E | Y | Y |
| 33 | 529 | E | Y | Y |
| 34 | 701 | A2 | Y | N |
| 35 | 728 | A1 | N | N |
| 36 | 902 | E | Y | Y |
| 37 | 902 | E | Y | Y |
| 38 | 903 | E | Y | Y |
| 39 | 903 | E | Y | Y |
| 40 | 1030 | A1 | N | Y |
| 41 | 1036 | A2 | N | N |
| 42 | 1330 | E | Y | Y |
| 43 | 1330 | E | Y | Y |
| 44 | 1331 | E | Y | Y |
| 45 | 1331 | E | Y | Y |

Table S4 the band gap, SHG coefficient, refractive index and birefringence (Δn) @ 1064nm under various temperature.

| T(K) | Band gap (eV) | d_16_(-d_22_),pm/V | n_e_ | n_o_ | Δn @1064nm |
| --- | --- | --- | --- | --- | --- |
| 13 | 7.81 | 0.87 | 1.6430 | 1.6000 | 0.0430 |
| 20 | 7.81 | 0.87 | 1.6429 | 1.5999 | 0.0430 |
| 40 | 7.81 | 0.87 | 1.6429 | 1.6000 | 0.0429 |
| 60 | 7.81 | 0.87 | 1.6429 | 1.6001 | 0.0428 |
| 80 | 7.81 | 0.87 | 1.6429 | 1.6000 | 0.0429 |
| 100 | 7.81 | 0.87 | 1.6428 | 1.5998 | 0.0430 |
| 120 | 7.80 | 0.87 | 1.6427 | 1.5998 | 0.0429 |
| 140 | 7.80 | 0.87 | 1.6425 | 1.5996 | 0.0429 |
| 160 | 7.79 | 0.87 | 1.6423 | 1.5994 | 0.0429 |
| 180 | 7.79 | 0.87 | 1.6420 | 1.5990 | 0.0430 |
| 200 | 7.78 | 0.87 | 1.6417 | 1.5988 | 0.0429 |
| 220 | 7.78 | 0.87 | 1.6414 | 1.5985 | 0.0429 |
| 240 | 7.77 | 0.87 | 1.6411 | 1.5982 | 0.0429 |
| 260 | 7.77 | 0.87 | 1.6407 | 1.5978 | 0.0429 |
| 280 | 7.76 | 0.87 | 1.6405 | 1.5976 | 0.0429 |
| 300 | 7.75 | 0.87 | 1.6401 | 1.5972 | 0.0429 |
